# Circulating Tumor Cells: From the Laboratory to the Cancer Clinic

**DOI:** 10.3390/cancers12103065

**Published:** 2020-10-20

**Authors:** Noriyoshi Sawabata

**Affiliations:** Department of Thoracic and Cardio-Vascular Surgery, Nara Medical University School of Medicine, 840 Shijo-cho, Kashihara 634-8552, Japan; nsawabata@naramed-u.ac.jp; Tel.: +81-744-22-3051

**Keywords:** circulating tumor cell, laboratory, cancer clinic

Circulating tumor cells (CTCs) are cells that are separated from the primary tumor, move through the bloodstream, and spread from the original tumor to other sites, causing cancer metastasis. CTCs are present in the peripheral blood of cancer patients, and their detection helps in elucidating the process of metastasis. Unlike other blood cells, the number of blood CTCs is very low, and they are difficult to detect. Morphologically, CTCs are of two types: singular CTCs and clustered CTCs. The potential for persistent existence is greater in clustered CTCs, which is a predictor of poor prognosis [1,2]. Clinical biomarkers such as tumor markers and features of radiological diagnosis are surrogate markers for detecting clustered CTCs [2]. It is well known that cancer metastasis often occurs via clustered CTCs [3], and they have a metastatic nest formation ability that is more than 10 times higher than that of a singular CTC [4].

In the past, metastasis was thought to be caused by tumor cells that are transformed into the single-shot mesothelium type due to epithelial–mesenchymal transition (EMT) [5]. CTCs that become resistant to drug treatment certainly have a very high appearance of markers in the mesenchymal system, and they show an epithelium appearance [6]. This phenomenon was difficult to explain by previous concepts. Therefore, the concept of hybrid EMT was proposed. Its characteristics are as follows: (1) the cluster-forming ability of CTCs is high [7,8], (2) they survive for a long period of time [9], (3) they have the characteristics of stem cells [10], (4) they have a high tumor-forming ability [11], and (5) they remain in a cluster while moving in capillaries [12]. A study explored CTC morphology by amplifying CTCs by short-term culture and revealed that tight clusters are less likely to appear depending on the therapy and that the prognosis of patients with tight clusters is poor [13]. In another study using real-time polymerase chain reaction (rt-PCR) to explore the DNA characteristics of CTC clusters, it was found that the frequency of the DNA appearance of the epithelial system was low, and the frequency of the DNA appearance of the mesenchymal system was high [14].

A singular CTC often undergoes apoptosis when it is away from the tumor’s microenvironment, which is unlikely to lead to recurrence [4]. Clustered CTCs, on the other hand, have stem cell-like elements that make them unlikely to undergo anoikis, and they therefore circulate throughout the body for a long period of time [6,7,8,9,10,11,12]. It is thought that CTCs become a recurrent lesion when they are deposited in a microenvironment that is suitable for tumor formation. Thus, the clinical implications of CTCs vary by diagnosis (cytology), the monitoring of therapy (cell counts and morphology), and the providing of samples for molecular diagnosis (next-generation sequencing: NGS; immunohistochemistry: IHC, if scant; amplification by short-term culture). CTC extraction and identification methods include size-selection methods such as chips and meshes, specific gravity methods, positive selection methods using epidermal antigens as indicators, and gene detection methods such as RT-PCR, etc. [15], which could be selected appropriately in order to take the assessment of CTCs from the laboratory to the cancer clinic.

The exploration of CTCs shall contribute to the improvement of cancer treatment.
